# HigB Reciprocally Controls Biofilm Formation and the Expression of Type III Secretion System Genes through Influencing the Intracellular c-di-GMP Level in *Pseudomonas aeruginosa*

**DOI:** 10.3390/toxins10110424

**Published:** 2018-10-24

**Authors:** Yueying Zhang, Bin Xia, Mei Li, Jing Shi, Yuqing Long, Yongxin Jin, Fang Bai, Zhihui Cheng, Shouguang Jin, Weihui Wu

**Affiliations:** 1 State Key Laboratory of Medicinal Chemical Biology, Key Laboratory of Molecular Microbiology and Technology of the Ministry of Education, Department of Microbiology, College of Life Sciences, Nankai University, Tianjin 300071, China; 2120160942@mail.nankai.edu.cn (Y.Z.); xbwwsq@mail.nankai.edu.cn (B.X.); shijing0213@mail.nankai.edu.cn (J.S.); 1120150348@mail.nankai.edu.cn (Y.L.); yxjin@nankai.edu.cn (Y.J.); baifang1122@nankai.edu.cn (F.B.); zhihuicheng@nankai.edu.cn (Z.C.); 2 Meishan Product Quality Supervision and Inspection Institute and National Pickle Quality Inspection Center, Meishan 620000, China; lmlovejn@163.com; 3 Department of Molecular Genetics and Microbiology, College of Medicine, University of Florida, Gainesville, FL 32610, USA; nksjin@nankai.edu.cn

**Keywords:** toxin-antitoxin system, HigB/HigA, *Pseudomonas aeruginosa*, type III secretion system, biofilm, c-di-GMP

## Abstract

Toxin-antitoxin (TA) systems play important roles in bacteria persister formation. Increasing evidence demonstrate the roles of TA systems in regulating virulence factors in pathogenic bacteria. The toxin HigB in *Pseudomonas aeruginosa* contributes to persister formation and regulates the expression of multiple virulence factors and biofilm formation. However, the regulatory mechanism remains elusive. In this study, we explored the HigB mediated regulatory pathways. We demonstrate that HigB decreases the intracellular level of c-di-GMP, which is responsible for the increased expression of the type III secretion system (T3SS) genes and repression of biofilm formation. By analyzing the expression levels of the known c-di-GMP metabolism genes, we find that three c-di-GMP hydrolysis genes are up regulated by HigB, namely PA2133, PA2200 and PA3825. Deletion of the three genes individually or simultaneously diminishes the HigB mediated regulation on the expression of T3SS genes and biofilm formation. Therefore, our results reveal novel functions of HigB in *P. aeruginosa*.

## 1. Introduction

Toxin-antitoxin (TA) systems have been found in almost all bacterial species [1]. A typical TA system is composed of a stable toxin and a labile antitoxin. Degradation of an antitoxin results in the activation of its cognate toxin, which inhibits bacterial growth by interfering with biosynthesis of macromolecules, cell division or reducing membrane potential [2]. The growth arrest facilitates bacterial survival under environmental stresses such as antibiotic treatment, nutrient deficiency and host immune clearance [3].

Based on the property of the antitoxin and the inhibitory mechanism of antitoxin on the cognate toxin, TA systems are classified into six groups (types I to VI). The type II TA systems are widely distributed in bacteria and archaea, and are composed of two proteins that are encoded in a bicistronic operon. Most type II toxins are endonucleases, the activities of which are repressed by the cognate antitoxins through direct interactions. Meanwhile, the antitoxins directly repress the expression of their own operons by binding to the promoter regions. Cross interactions between different TA systems have been reported [4,5]. Besides arresting bacterial growth, some type II TA systems have been found to influence virulence gene expression and biofilm formation [6,7,8,9,10]. 

*P. aeruginosa* is an opportunistic pathogen. It harbors various virulence factors and causes various infections in immunocompromised patients. The type III secretion system (T3SS) and biofilm formation have been demonstrated to play important roles in acute and chronic infections, respectively. In *P. aeruginosa*, the T3SS and biofilm formation are reciprocally regulated by a small RNA-protein (RsmY/Z-RsmA) mediated regulatory pathway and the secondary messenger molecule c-di-GMP [11]. 

At least 4 type II TA systems have been identified in *P. aeruginosa*, namely ParD/ParE, HicA/HicB, RelE/RelB and HigB/HigA [3,10,12,13,14]. Studies in *Proteus vulgaris* reveal that HigB cleaves mRNA with a preference in A rich sequences [15]. Wood et al found that the HigB/HigA of *P. aeruginosa* influences several virulence factors, including pyochelin and pyocyanin as well as swarming motility and biofilm formation [10]. We previously demonstrated that the *higB*/*higA* operon is induced by treatment with ciprofloxacin and HigB contributes to persister formation and controls the expression of T3SS genes [3]. However, the mechanism of HigB mediated regulation on the T3SS and biofilm formation remains unknown. 

In this study, we found that HigB influences T3SS and biofilm formation through c-di-GMP. We further identified the HigB regulated c-di-GMP metabolism genes. 

## 2. Results

### 2.1. HigB Represses Biofilm Formation

**Figure 1:**
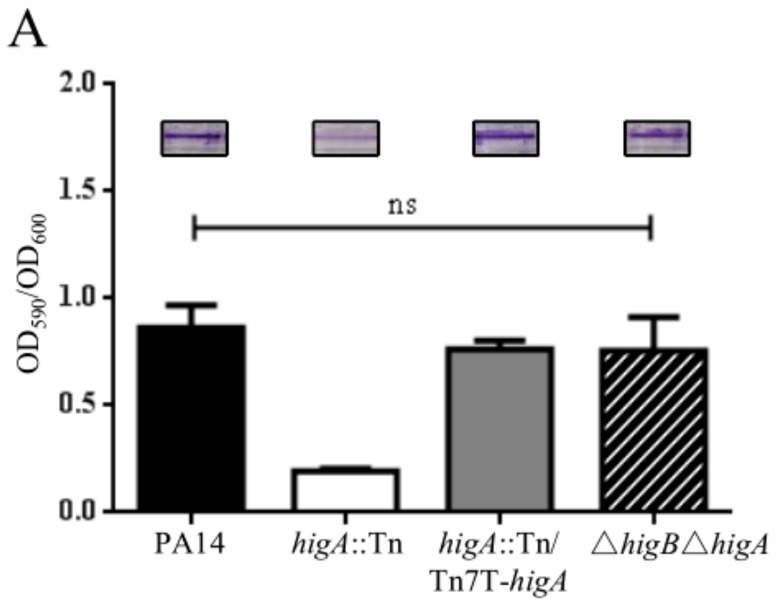

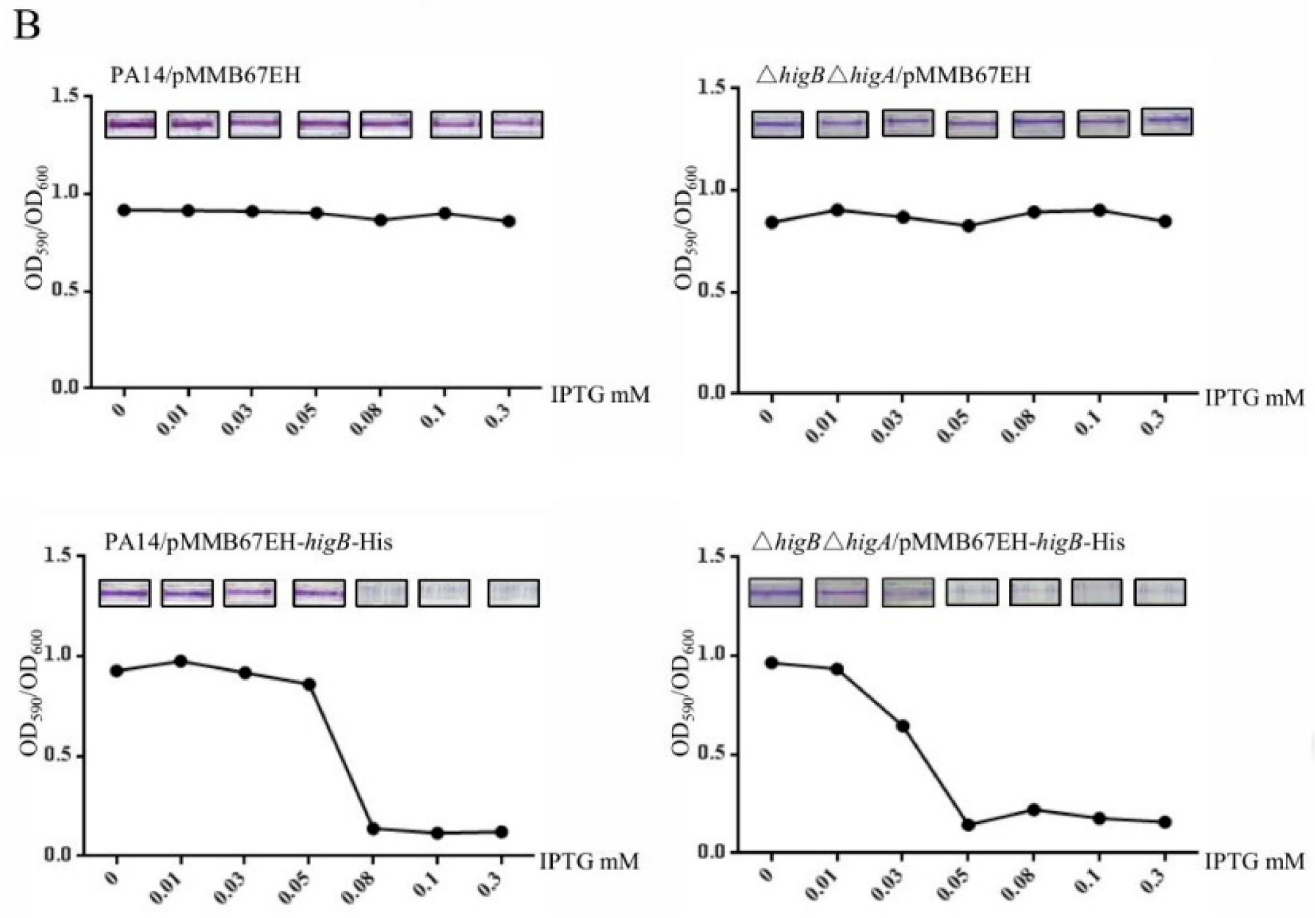
HigB affects biofilm formation. Bacteria were adjusted to an OD_600_ of 0.025 in LB broth and inoculated in each well of a 96-well plate. The plates were incubated at 37 °C for 24 h. The biofilm was visualized and quantified by crystal violet staining. (**A**) Biofilm formation by wild type PA14, the *higA*::Tn, the *higA* complemented strain and the Δ*higB*Δ*higA* mutant. ns, not significant. (**B**) Biofilm formation by PA14 or the Δ*higB*Δ*higA* mutant containing the empty vector pMMB67EH or the *higB* overexepression plasmid pMMB67EH-*higB*-His. IPTG was supplemented at the indicated concentrations.

Previously, Wood et al. demonstrated reduction of biofilm formation in a *higA* mutant, indicating a role of HigB in the repression of biofilm formation [10]. To verify the role of HigB in biofilm formation, we constructed a Δ*higB*Δ*higA* double mutant, which displayed normal biofilm formation (Figure 1A). In addition, overexpression of *higB* in wild type PA14 reduced biofilm formation (Figure 1B). These results suggest that HigB contributes to the repression of biofilm formation. 

### 2.2. HigB Controls the Intracellular c-di-GMP Level

**Figure 2:**
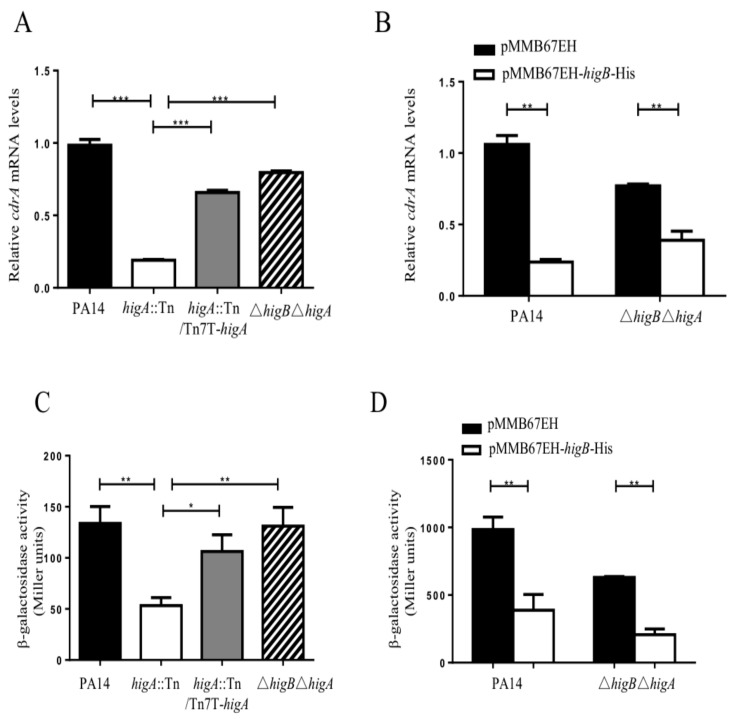
Role of HigB in the expression of *cdrA*. (**A**) PA14, the *higA*::Tn mutant, the complemented strain and the Δ*higB*Δ*higA* double mutant were grown to an OD_600_ of 2.0. The mRNA levels of *cdrA* were determined by real time PCR. (**B**) PA14 or the Δ*higB*Δ*higA* mutant containing pMMB67EH or pMMB67EH-*higB*-His was grown in the presence of 0.1 mM IPTG. The mRNA level of *cdrA* were determined by real time PCR. The ribosomal protein encoding gene *rpsL* was used as the internal control. Error bars represent the standard errors. ** *p* < 0.01, *** *p* < 0.005 by Student’s *t*-test. (**C**,**D**) Expression levels of the transcriptional fusion of P*_cdrA_*-*lacZ* were determined in PA14, the *higA*:Tn mutant, the complemented strain and the Δ*higB*Δ*higA* double mutant (**C**) or PA14 and the Δ*higB*Δ*higA* mutant containing pMMB67EH or pMMB67EH-*higB*-His grown in the presence of 0.1 mM IPTG (**D**). * *p* < 0.05, ** *p* < 0.01 by Student’s *t*-test.

**Figure 3:**
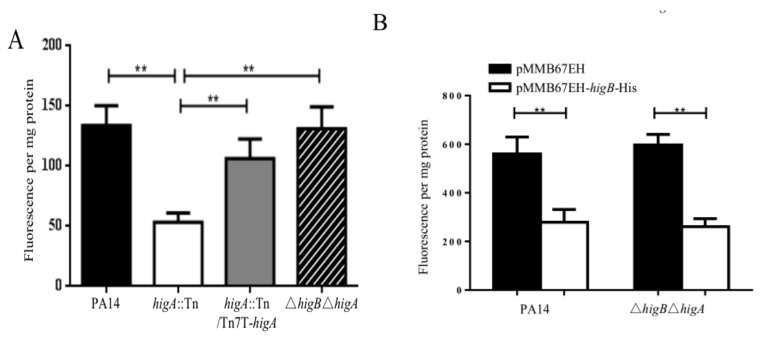
HigB affects the intracellular c-di-GMP levels. The intracellular levels of c-di-GMP in PA14, the *higA*::Tn mutant, the complemented strain and the Δ*higB*Δ*higA* double mutant (**A**) or PA14 and the Δ*higB*Δ*higA* mutant containing pMMB67EH or pMMB67EH-*higB*-His grown in the presence of 0.1 mM IPTG (**B**) were determined by thiazole orange based fluorescence. The bacteria were grown in LB to an OD _600_ of 2.0. The bacteria were lysed and the supernatant was incubated with thiazole orange to determine the c-di-GMP levels. **, *p* < 0.01 by Student’s *t*-test.

We previously found that HigB activates the expression of T3SS genes. In *P. aeruginosa*, the small RNAs RsmY/RsmZ and the secondary messenger molecule c-di-GMP reciprocally control T3SS and biofilm formation [16]. Therefore, we examined whether the HigB-HigA TA module influences the levels of RsmY/RsmZ and c-di-GMP. Neither mutation of *higA* nor overexpression of *higB* resulted in alteration of RsmY/RsmZ levels (Figure S1). Then, we examined c-di-GMP levels. The transcription of *cdrA* is controlled by the intracellular level of c-di-GMP [17]. Thus, the *cdrA* expression level has been used as a c-di-GMP indicator [17]. Mutation of *higA* or overexpression of *higB* reduced the mRNA level of *cdrA* (Figure 2A,B). To examine whether the reduction of *cdrA* mRNA is due to lower promoter activity, we constructed a transcriptional fusion of the *cdrA* promoter and a *lacZ* gene (P*_cdrA_*-*lacZ*). The expression of LacZ was lower in the *higA* mutant or the *higB* overexpression strain (Figure 2C,D), indicating a lower c-di-GMP level. Furthermore, a thiazole orange-based fluorescent assay [18] revealed lower c-di-GMP levels in the *higA* mutant and the strain overexpressing *higB* (Figure 3A,B). These results demonstrate that HigB represses the bacterial c-di-GMP level. 

### 2.3. HigB Reciprocally Controls Biofilm Formation and the T3SS by Influencing c-di-GMP Levels 

**Figure 4:**
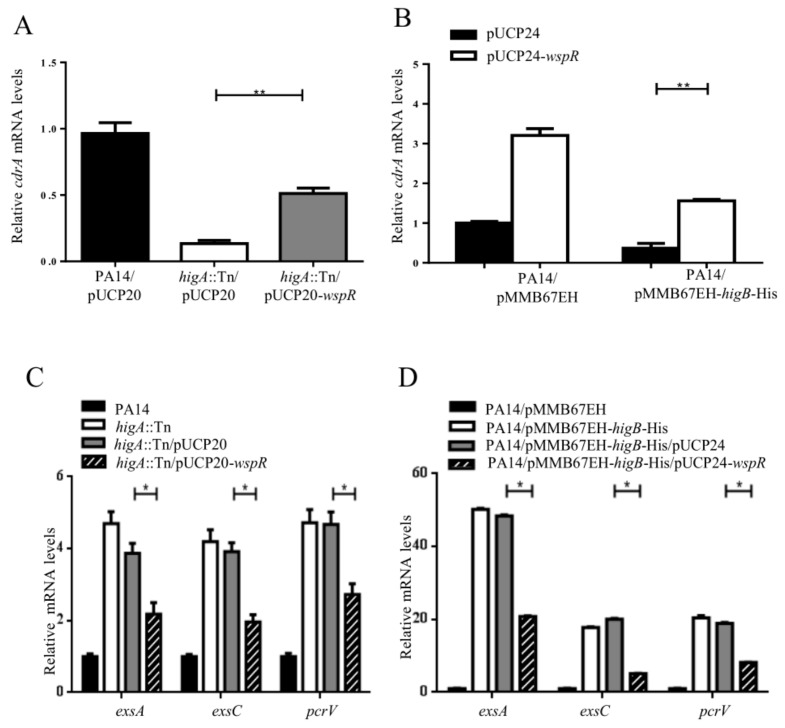
Role of c-di-GMP in HigB mediated regulation on biofilm formation and T3SS gene expression. (**A**) The mRNA levels of *cdrA* in PA14 and the *higA* mutant carrying the empty vector pUCP20 or the *higA* mutant carrying pUCP20–*wspR*. (**B**) The mRNA levels of *cdrA* in PA14 carrying the empty vector pUCP24 or pUCP24–*wspR* together with pMMB67EH or pMMB67EH-*higB*-His. The bacteria were grown in the presence of 0.1 mM IPTG. (**C**) The mRNA levels of the T3SS genes, *exsA*, *exsC* and *pcrV* in PA14 and the *higA* mutant carrying pUCP20 or the *higA* mutant carrying pUCP20–*wspR*. (**D**) PA14 carrying the empty vector pUCP24 or pUCP24–*wspR* together with pMMB67EH or pMMB67EH-*higB*-His were grown in the presence of 0.1 mM IPTG. The mRNA levels of *exsA*, *exsC* and *pcrV* were determined by real time PCR. * *p* < 0.05, ** *p* < 0.01 by Student’s *t*-test.

To examine whether the reduced c-di-GMP level is responsible for the HigB mediated repression of biofilm formation and activation of the T3SS genes, we overexpressed a diguanylate cyclases WspR, which has been shown to increase the intracellular c-di-GMP level and enhance biofilm formation [19,20,21]. In the *higA* mutant or the *higB* overexpressing strain, overexpression of *wspR* increased the expression level of *cdrA*, indicating an increase of the intracellular level of c-di-GMP (Figure 4A,B). Consistently, overexpression of *wspR* restored the levels of biofilm formation and down regulated the T3SS genes in the *higA* mutant or the *higB* overexpressing strain. (Figure 4C,D).

### 2.4. Identification of c-di-GMP Metabolism Genes that Are Controlled by HigB

**Figure 5:**
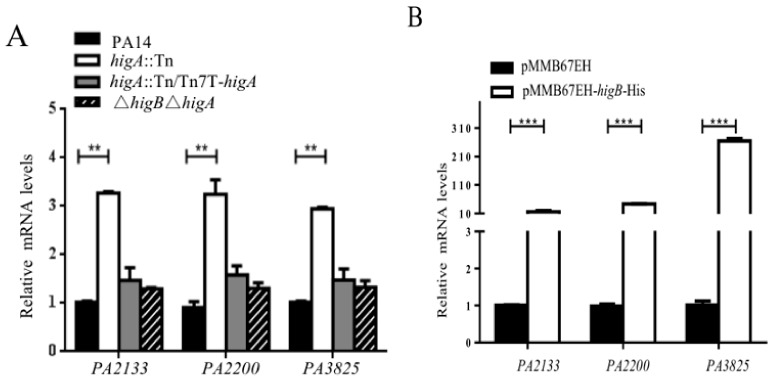
HigB up-regulates the expression of *PA2133*, *PA2200* and *PA3825.* (**A**) PA14, the *higA*:Tn mutant, the complemented strain and the Δ*higB*Δ*higA* double mutant were grown to an OD_600_ of 2.0. The mRNA levels of *PA2133*, *PA2200* and *PA3825* were determined by real-time PCR. (**B**) PA14 containing pMMB67EH or pMMB67EH-*higB*-His was grown in the presence of 0.1 mM IPTG. The mRNA levels of *PA2133*, *PA2200* and *PA3825* were determined by real-time PCR. ** *p* < 0.01, *** *p* < 0.005 by Student’s *t*-test.

In *P. aeruginosa*, there are at least 40 genes that are involved in the metabolism of c-di-GMP [22]^.^ Diguanylate cyclases (DGCs), containing a GGDEF motif, synthesize c-di-GMP. Phosphodiesterases (PDEs), containing a HD-GYP or EAL domain, degrade c-di-GMP [22]. To understand the mechanism of HigB mediated repression on the intracellular c-di-GMP level, we compared the mRNA levels of the 40 c-di-GMP metabolism genes between PA14 and the *higA* mutant. Among the diguanylate cyclases, PA3343 and PA5487 were up regulated in the *higA* mutant (Figure S2A), which should not lead to the reduction of c-di-GMP. PA0861, PA1727 and PA2567 were up regulated in the *higA* mutant (Figure S2B). However, since they contain both the GGDEF and EAL motifs, their roles in controlling the c-di-GMP level remain elusive. Of note, among the phosphodiesterases, three genes, namely PA2133, PA2200 and PA3825 were up regulated the most (≥3-fold) in the *higA* mutant (Figure S2C). All three genes contain an EAL domain [2]. Complementation with a *higA* gene reduced the expression of those genes (Figure 5A). Overexpression of HigB also increased the expression of these genes (Figure 5B). These results indicate that the three genes might be involved in the HigB mediated repression of the intracellular c-di-GMP level. 

### 2.5. PA2133, PA2200 and PA3825 Contribute to the HigB Mediated Decrease of c-di-GMP 

**Figure 6:**
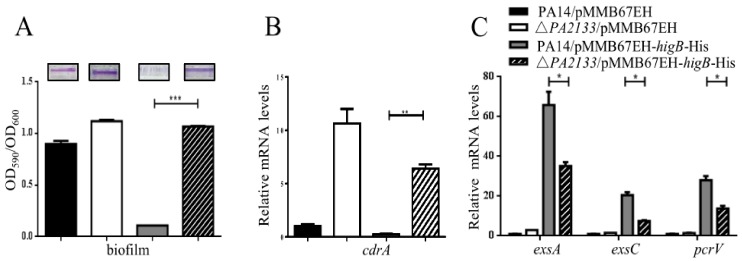

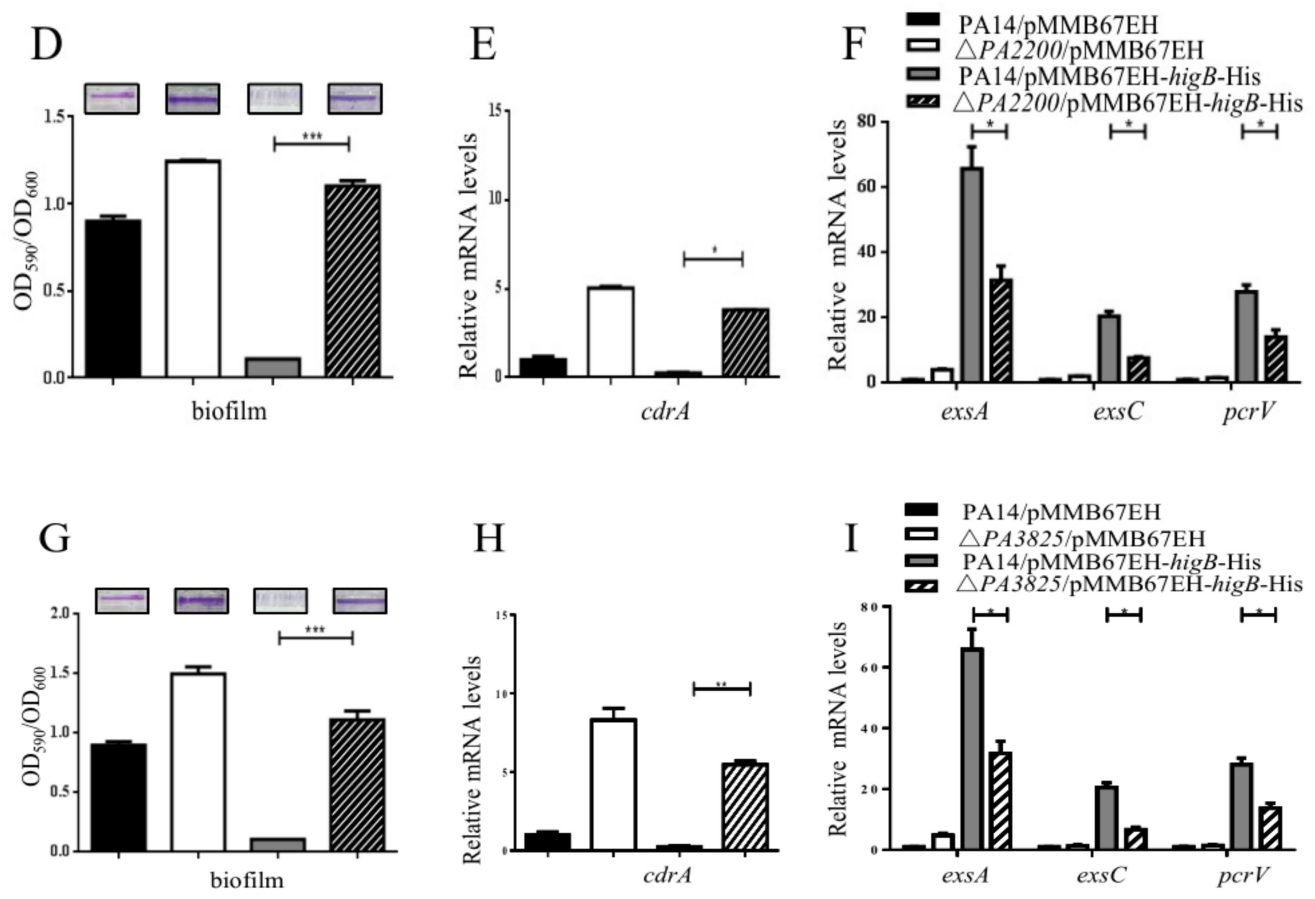
Roles of PA2133, PA2200 and PA3825 in HigB mediated regulation on biofilm formation and the expression of *cdrA* and T3SS genes. Biofilm formation, mRNA levels of *cdrA* and the T3SS genes were examined in PA14 and the indicated mutant containing the empty vector pMMB67EH or the *higB* overexpression plasmid pMMB67EH-*higB*-His. Biofilm formation was observed and quantified by staining with crystal violet. The mRNA levels of indicated genes were examined by real-time PCR. Comparison between PA14 and the Δ*PA2133* (**A**–**C**), the Δ*PA2200* (**D**–**F**) and the Δ*PA3825* mutants (**G**–**I**). Error bars represent the standard errors. * *p* < 0.05, ** *p* < 0.01, *** *p* < 0.005 by Student’s *t*-test.

**Figure 7:**
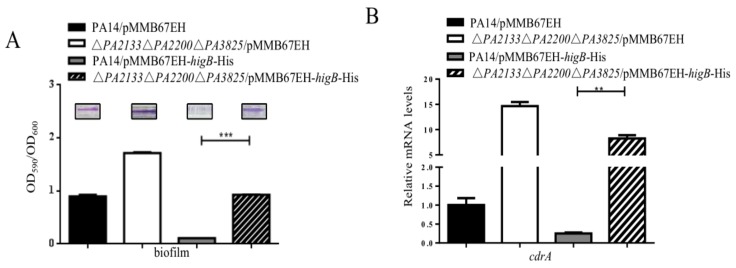

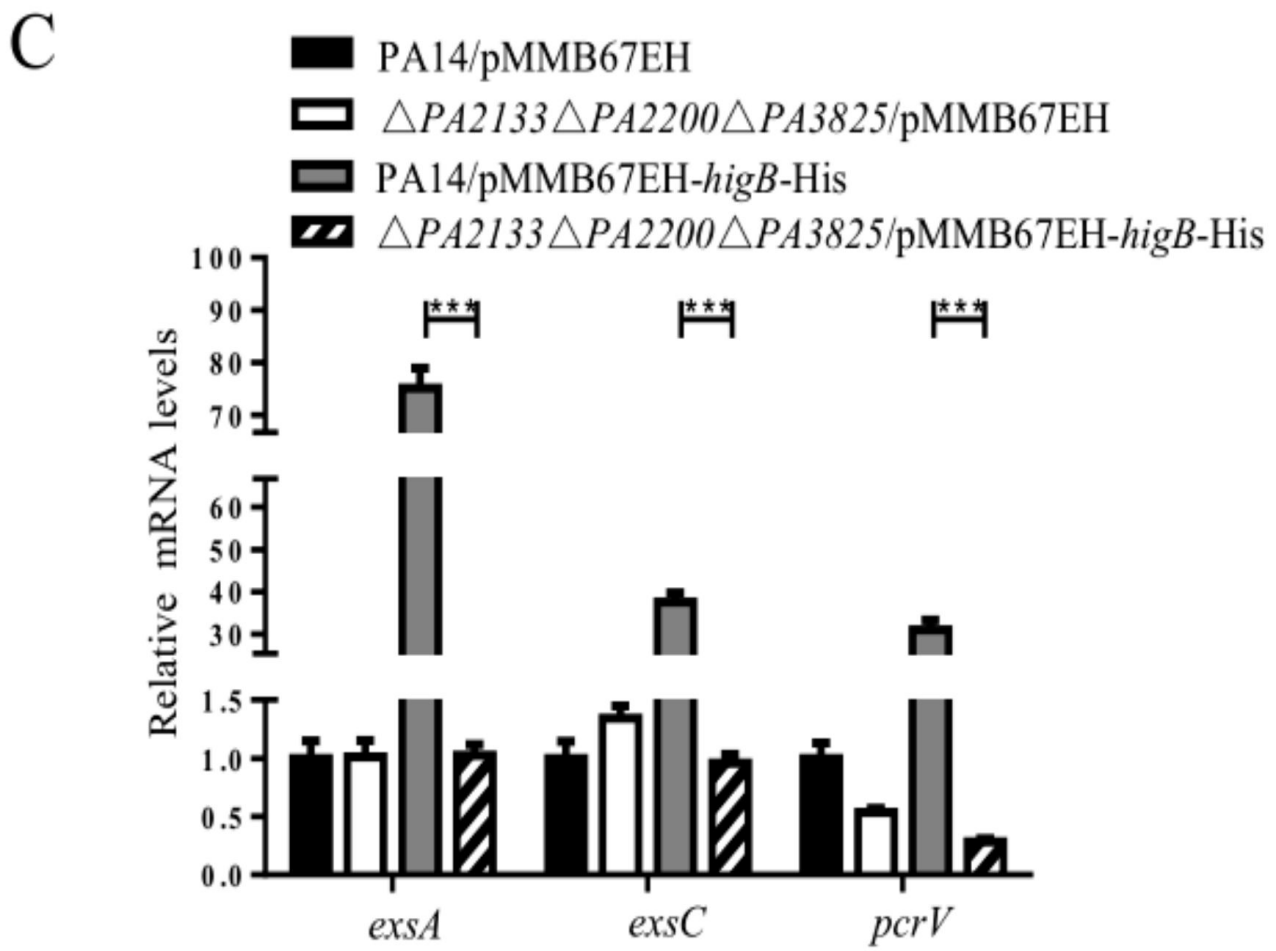
Effects of simultaneous deletion of PA2133, PA2200 and PA3825 on HigB mediated regulation on biofilm formation, *cdrA* and T3SS gene expression. (**A**) Biofilm formation by PA14 and the Δ*PA2133*Δ*PA2200*Δ*PA3825* mutant containing the empty vector pMMB67EH or pMMB67EH-*higB*-His. IPTG was supplemented at 0.1 mM. (**B**) PA14 or the Δ*PA2133*Δ*PA2200*Δ*PA3825* mutant containing pMMB67EH or pMB67EH-*higB*-His was grown to an OD_600_ of 2.0 in the presence of 0.1 mM IPTG. The mRNA level of *cdrA* (**C**) and *exsA, exsC* and *pcrV* were determined by real time PCR. Error bars represent the standard errors. ** *p* < 0.01, *** *p* < 0.005 by Student’s *t*-test.

To explore the roles of PA2133, PA2200 and PA3825 in the reduction of c-di-GMP, each individual gene was knocked out in wild type PA14. Compared to wild type PA14, mutation of each of the genes increased the biofilm formation and the expression of *cdrA* as well as partially reduced the expression of T3SS genes upon overexpression of *higB* (Figure 6). Then we constructed a triple mutant by deleting all the three genes, resulting in ΔPA2133ΔPA2200ΔPA3825. Upon overexpressing *higB*, the triple mutant displayed similar levels of biofilm formation, expression of *cdrA* and the T3SS genes as those in wild type PA14 (Figure 7). These results suggest that the up regulation of PA2133, PA2200 and PA3825 are involved in the HigB mediated reduction of intracellular level of c-di-GMP and the reciprocal regulation of the T3SS genes and biofilm formation.

## 3. Discussion

In this study, we found that HigB controls the intracellular c-di-GMP level in *P. aeruginosa*. Studies on *P. vulgaris* HigB demonstrate that the toxin cleaves mRNA in a ribosome dependent manner with a preference of A rich sequences [15]. Overexpression of the *Mycobacterium tuberculosis higB* gene resulted in the down regulation of IdeR and Zur regulated genes. In addition, the *M. tuberculosis* HigB cleaves tmRNA, which is involved in ribosome rescue [23,24]. The antitoxin HigA of *M. tuberculosis* has been confirmed to bind to the promoter of the *higB*-*higA* operon, however, no other binding site of HigA was identified on the *M. tuberculosis* chromosome [25]. These results suggest that HigB might specifically regulate a subset of genes in *M. tuberculosis*. Studies in *Caulobacter crescentus* revealed that HigB controls the bacterial tolerance to nalidixic acid and cell cycle by targeting the mRNAs of *acrB2* and *ctrA*, respectivel and that the preferred cleavage site of HigB was found to be UCG [26]. Considering the ribosome (translation) dependent cleavage by HigB and the possible random distribution of the A rich coding sequences or UCG, ribosome stalling and the sequences adjacent to HigB preferred regions might be involved in the cleavage specificity and efficiency of HigB. 

In *P. aeruginosa*, HigB controls a subset of virulence factors, including pyochelin, pyocyanin, T3SS, swarming motility and biofilm formation [3,10]. The amino acid residues G17, I66, W72 and R73 of HigB are conserved in various species and might be the active site. Point mutation of each of the residues reduced the growth inhibitory effect of HigB (Figure S3). We further found that HigB represses the intracellular c-di-GMP level though up regulation of the expression of three c-di-GMP degradation genes, namely PA2133, PA2200 and PA3825. Based on the endonuclease function of HigB, we suspect that HigB might repress the expression of the negative regulator(s) of those three genes. Further studies are needed to elucidate this aspect of the regulatory mechanism.

Recently, PA2133 has been demonstrated to control biofilm formation through degrading c-di-GMP [27]. However, PA2133 represses the expression of T3SS genes under a T3SS inducing condition independent of its phosphodiesterase activity [27]. In our study, we found that mutation of PA2133 in PA14 did not affect the expression of the T3SS genes under a T3SS non-inducing condition (grown in LB), but reduced HigB mediated up regulation of the T3SS genes. Therefore, PA2133 might possess dual functions in controlling c-di-GMP and the T3SS. 

Overall, we found that HigB represses biofilm formation through reducing the intracellular c-di-GMP level. Previous studies demonstrate enhanced persister cell formation in biofilm [28,29]. In *P. aeruginosa*, HigB promoted persister cells might be defective in biofilm formation. We suspect that the cells with activated HigB might be embedded in the extracellular matrix generated by other cells, thus forming a heterogeneous community that is highly tolerant to antibiotics and host immune attack. 

## 4. Materials and Methods

### 4.1. Strains, Plasmids and Growth Conditions

The bacterial strains, plasmids and primers used in this study are listed in Table S1. The *Escherichia coli* and *P. aeruginosa* strains were cultured in the Luria–Bertani (LB) broth (10 g/L tryptone, 5 g/L yeast extract, 5 g/L NaCl, pH 7.4) at 37 °C. When needed, antibiotics were used at the following concentrations (μg/mL): For *E.coli*, tetracycline 100 ampicillin 100; for *P. aeruginosa*, gentamicin 100, carbenicillin 150 (BBI life sciences). Isopropyl β-d-1-thiogalactopyranoside (IPTG) was added at indicated concentrations. Chromosomal gene mutation was constructed as described previously [30].

### 4.2. RNA Preparation and qRT-PCR Analysis

RNA was purified with a RNAprep Kit (Tiangen Biotech, Beijing, China). Briefly, bacteria were grown to the stationary phase (OD_600_ = 2). 200 μL bacterial culture was collected, followed by RNA purification. cDNA was synthesized from each RNA sample by using a Prime-Script Reverse Transcriptase (TaKaRa, Dalian, China). Real-time PCR was performed with the SYBR Premix ExTaq II (TaKaRa) in a CFX Real-Time system (Bio-Rad, Hercules, CA, USA). *rpsL* (the 30s ribosomal protein coding gene) was used as the internal control.

### 4.3. Biofilm Assays

Biofilm formation was examined as previously described [31]. Briefly, bacteria were grown to an OD_600_ of 0.8. Then the bacteria were diluted to an OD_600_ of 0.025 in LB broth and 200 μL of the bacterial suspension was inoculated in each well of a 96-well plate. The plates were incubated at 37 °C for 24 h. Then the wells were washed with 1× PBS (137 mM NaCl, 2.7 mM KCl, 10 mM Na_2_HPO_4_, pH 7.4) 3 times and dried in an incubator for 20 min. The wells were stained with 0.05% crystal violet for 20 min. Afterwards, the wells were washed twice with PBS. 200 μL methanol was added to each well and incubated at room temperature for 15 min. The solubilized crystal violet and the cell density were quantified by measuring its optical density (OD) at 590 nm and 600 nm with the microplate reader Varioskan Flash (Thermo Scientific, Billerica, MA, USA).

### 4.4. Quantification of c-di-GMP by Thiazole Orange

The concentration of c-di-GMP was measured as described previously [18]. Bacteria at the OD_600_ of 2.0 were harvested by centrifugation at 13,000× *g* for 3 min. The pellet was resuspended in 1 mL 10 mM Tris-HCl (pH 8.0) with 100 mM NaCl. The cells were lysed through sonication. The protein concentrations were measured with a Bio-Rad Protein Assay Dye Reagent Concentrate (Bio-Rad, Hercules, CA, USA). 12% perchloric acid was then added to the mixture and incubated for 10 min on ice. The solution was neutralized by aqueous alkali (0.4 M Tris, 3 M KOH and 2 M KCl) [32]. The solution was subjected to centrifugation at 12,000× *g* for 5 min at 4 °C. The supernatant was then filtered through a 0.2 μm filter and a 3 kD exclusion column. The supernatant was heated at 95 °C for 5 min and then cooled to room temperature for 15 min, followed by addition of thiazole orange (TO) at a final concentration of 30 μM. The mixture was incubated for 12 h at 4 °C. The signal was detected with a Varioskan Flash microplate reader (Thermo Scientific, Billerica, MA, USA) at 10 °C with the following settings: excitation 508 nm, emission 533 nm [18,32].

### 4.5. Transcriptional Reporter Assay

To construct the *cdrA* promoter and *lacZ* transcriptional fusion (P*_cdrA_*-*lacZ*), a DNA fragment containing the *cdrA* promoter was amplified by PCR, and cloned upstream of the promoter-less *lacZ* in the plasmid pDN19*lacZ*Ω. The empty vector pDN19*lacZ*Ω was used as a negative control. To measure the LacZ levels, overnight bacterial cultures were diluted at 1:100 in fresh LB and grown to an OD_600_ of 2.0. The β-galactosidase activity was determined with ortho-nitrophenyl-galactopyranoside (ONPG) (BBI life sciences) as described previously [33,34].

## Supplementary Materials

The following are available online at http://www.mdpi.com/2072-6651/10/11/424/s1, Figure S1: Role of HigB in the expression of *rsmY* and *rsmZ*, Figure S2: Expression levels of c-di-GMP metabolism genes in PA14 and the *higA* mutant, Figure S3: Effects of point mutations on the growth inhibitory function of HigB, Table S1: Bacterial strains, plasmids and primers used in this study.
