# Supplementary material for: HigB Reciprocally Controls Biofilm Formation and the Expression of Type III Secretion System Genes through Influencing the Intracellular c-di-GMP Level in Pseudomonas aeruginosa
Source: Toxins (Basel). 2018 Oct 24;10(11):424. doi: 10.3390/toxins10110424 (PMC6265988; doi:10.3390/toxins10110424)
Supplement: Supplementary file 1 [file toxins-10-00424-s001.pdf]

Supplementary Materials: HigB Reciprocally Controls Biofilm Formation and the Expression of Type III Secretion System Genes through Influencing the Intracellular c-di-GMP Level in *Pseudomonas aeruginosa*

Yueying Zhang, Bin Xia, Mei Li, Jing Shi, Yuqing Long, Yongxin Jin, Fang Bai, Zhihui Cheng, Shouguang Jin and Weihui Wu

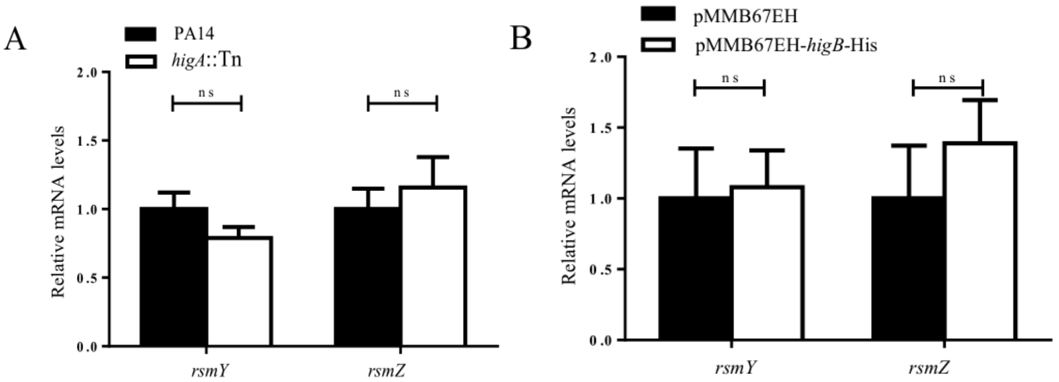

Figure S1. Role of HigB in the expression of *rsmY* and *rsmZ*. (**A**) Wild type PA14 and the *higA*::Tn mutant were grown to an OD_{600} of 2.0. The levels of *rsmY* and *rsmZ* were determined by real time PCR. (**B**) PA14 containing pMMB67EH or pMMB67EH-*higB* was grown to an OD_{600} of 2.0. The levels of *rsmY* and *rsmZ* were determined by real time PCR. ns, not significant.

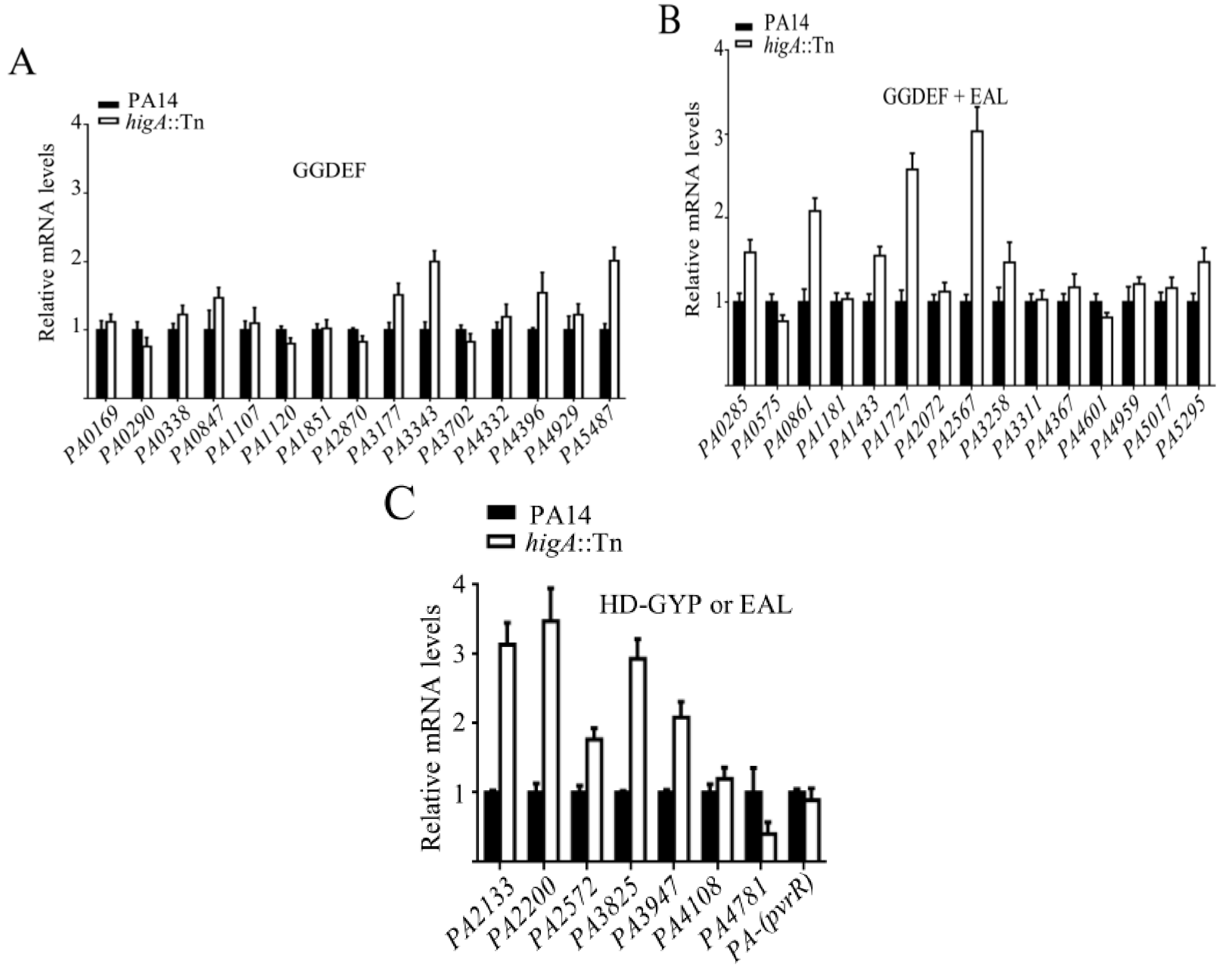

Figure S2. Expression levels of c-di-GMP metabolism genes in PA14 and the *higA* mutant. PA14 and the *higA*::Tn mutant were grown to an OD_{600} of 2.0. The mRNA level of relative genes were determined by real time PCR. (**A**) Genes containing the GGDEF domain. (**B**) Genes containing both the GGDEF and EAL domains. (**C**) Genes containing the HD-GYP or EAL domain.

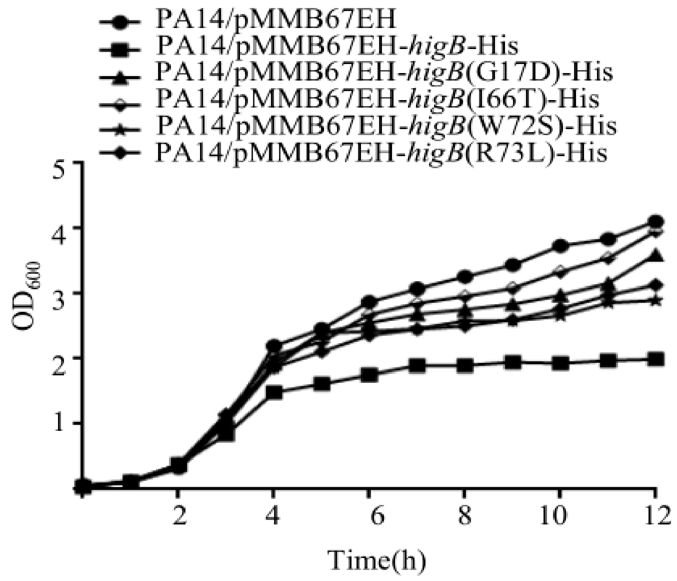

Figure S3. Effects of point mutations on the growth inhibitory function of HigB. PA14 containing the empty vector pMMB67EH, pMMB67EH carrying wild type *higB* or *higB* with indicated point mutations (G17D, I66T, W72S, R73L) were grown at 37 °C in LB medium in the presence of 0.1 mM IPTG. The bacterial density were monitored every hour for 12 hours.

Table S1. Bacterial strains, plasmids and primers used in this study.

Bacterial strain	**Description**	**Source (Reference)**
	P. aeruginosa	
PA14	Wild type strain of *Pseudomonas aeruginosa*	1
higA::Tn	PA14 with MAR2 × T7 transposon inserted at *higA*; Gm r	1
higA:Tn /Tn7T-*higA*	*higA*::Tn with *higA* inserted on chromosome with mini-Tn7T insertion; Tc r	This study
△*higB*△*higA*	PA14 deleted of *higB* and *higA*	This study
△*PA2133*	PA14 deleted of PA*2133*	This study
△*PA2200*	PA14 deleted of PA*2200*	This study
△*PA3825*	PA14 deleted of PA*3825*	This study
**Plasmid**	**Description**	**Source (Reference)**
pUCP20	*Escherichia–Pseudomonas* shuttle vector without *lac* promoter; Ap r	2
pEX18Tc	Gene replacement vector; Tc r	2
pMMB67EH	Expression vector with *tac* promoter; Ap r	3
pDN19lacΩ	Promoterless *lacZ* fusion vector; Sp r, Sm r, Tc r	4
pUC18T-mini-Tn7T-Tc	mini-Tn7 base vector from insertion into chromosome attTn7 site; Tc r	2
pUC18T-mini-Tn7T-Tc-*higA*	pUC18T-mini-Tn7T-Tc with *higA*; Tc r	This study
pEX18Tc-△*higB*△*higA*	*higB* and *higA* gene of PAK deletion on pEX18Tc; Tc r	This study
pMMB67EH-*higB* -His	*higB* gene with His-tag driven by *tac* promoter on pMMB67EH; Ap r	This study
pUCP20-*wspR*	*wspR* gene of PA14 gene on pUCP20; Ap r	This study
pUCP24-*wspR*	*wspR* gene of PA14 on pUCP24; Gm r	This study
**Primer**	**Sequence (5′→3′)**	**Function**
KpnⅠ-higB-F	CGGGGTACCAGTGAAGTTAACGCTTAACGTTAAG	*higB* cloning
HindIII-higB-R	CCCAAGCTTTCAGTGGTGGTGGTGGTGGTGACCTCCGTGGTAATCAACTATTTCGACTTC	*higB* cloning
EcoRI-wspR-F	GCAGAATTCATGCACAACCCTCATGAGAGCAAGA	*wspR* cloning
HindIII--wspR-R	ATAAAGCTTTCAGCCCGCCGGGGCCGGCGGCACC	*wspR* cloning
SacI-higBhigAup-F	TCGATGGAGCTCTAGCGGATGGTGGGGAAGGG	*higB* and *higA* deletion
KpnI-higBhigAup-R	CGGGGTACCATGCCCCGCTCCATCCCTTC	*higB* and *higA* deletion
KpnI-higBhigAdown-F	CGGGGTACCCGGTGACGTTGATCGTAGAGCCC	*higB* and *higA* deletion
HindIII-higBhigAdown-R	CCCAAGCTTCATCCCCCACTTCACCGAGGG	*higB* and *higA* deletion
BamHI-Pro-cdrA-F	CGGACGGGATTCGAAAATCTCCCTATCTGCGT	*cdrA* promoter cloning
EcoRI--Pro-cdrA-R	GGCGTGGGATTCCCATGGCAGTTGGCGACGAC	*cdrA* promoter cloning
EcoRI-2200up-F	CGAACGGAATTCGCCGGGTCTCATCCACCT	*PA2200* deletion
BamHI-2200up-R	GCGCTGGGATCCTTCATCTCGCGGAGCATC	*PA2200* deletion
BamHI-2200down-F	CCTGGAGGATCCCGTGGTCGTCGAAGGGAT	*PA2200* deletion
HindIII-2200down-R	GCCTGTAAGCTTCCAGGTCGCCGAGGTAAT	*PA2200* deletion

EcoRI-3825up-F	GCGTGCGAATTCGCTTCGTCTTCCGCATCG	*PA3825* deletion
SacI-3825up-R	ATCACCGAGCTCCAGCACGGACAACGACAGC	*PA3825* deletion
SacI-3825down-F	GAAGGGGAGCTCGCTTCCGTCGTCGCACTT	*PA3825* deletion
HindIII-3825down-R	CGGCCTAAGCTTCGGTTTTTTGTTGTCTGCGA	*PA3825* deletion
EcoRI-2133up-F	AACGGCGAATTCGGCGGCATCGACCTGTAC	*PA2133* deletion
BamHI-2133up-R	GCTCGTGGATCCGAGCCTGTGGGGAACCGT	*PA2133* deletion
BamHI-2133down-F	TCGATTGGATCCATACCCGCGAAGCACG	*PA2133* deletion
Hind-2133down-R	CTGAGGAAGCTTAACCAGCCGCTGATGTCC	*PA2133* deletion
cdrA-F	ATGTGAATCCGACTCTGA	RT-PCR
cdrA-R	CGTTGAACTGACTGTTGA	RT-PCR
pvrR-F	CATCAGTTGTATTGAGTTG	RT-PCR
pvrR-R	GAAGAAGATTCAGTTCCT	RT-PCR
exsA-F	GCTATGTCGTAAGTACCA	RT-PCR
exsA-R	GAAGCCTTGTAGAAACTG	RT-PCR
exsC-F	ATGGATTTAACGAGCAAGGTCAA	RT-PCR
exsC-R	GAGGGACAGGGAAGGCAAA	RT-PCR
pcrV-F	CACGCTCTATGGCTATGC	RT-PCR
pcrV-R	AAGGTATCCAGATTGCTCAG	RT-PCR
rpsL-F	TATGCCGTGTACGTCTGA	RT-PCR
rpsL-R	CACTACGCTGTGCTCTTG	RT-PCR
PA2133-F	CGTTGAACTGACTGTTGA	RT-PCR
PA2133-R	ACGTGCTTCGCGGGTATA	RT-PCR
PA3825-F	GAGCAAGTGGCGATCATG	RT-PCR
PA3825-R	AACAACGACGTGCAATAGAT	RT-PCR
PA2200-F	ATCTACGTGGACGATTTC	RT-PCR
PA2200-R	GATGGACTGAGTGAAGAC	RT-PCR
PA3343-F	TTCCAATCTCTATGCCGAC	RT-PCR
PA3343-R	GTAGTCACGCTCCAGTTC	RT-PCR
PA1107-F	TTCAACCTGATCCACATC	RT-PCR
PA1107-R	ATGGGCGTACATGATTTC	RT-PCR
PA4929-F	TACAACCTGTTCCTCTTC	RT-PCR
PA4929-R	CAGAAATACTCGAAACCAT	RT-PCR
PA4781-F	CCTACAGTCATCACGAAC	RT-PCR
PA4781-R	CTGGTCAGTTCATCGTAG	RT-PCR
PA0290-F	CAAGAAGCACATGGAGAG	RT-PCR
PA0290-R	AAGAAATAGCGACGGTTG	RT-PCR
PA3702-F	GAATACCTGGAGATGGAG	RT-PCR

PA3702-R	TTGTAGCTCTTGAAATAGTC	RT-PCR
PA3177-F	AAACCATCAAGGACCACC	RT-PCR
PA3177-R	ACAGCAGGACGAGGAATT	RT-PCR
PA2870-F	CGGAACTCATGCTCAAGG	RT-PCR
PA2870-R	TGTCGTTGATGTTCTTGAA	RT-PCR
PA1851-F	CTGAACCTGGCGGTATTC	RT-PCR
PA1851-R	GCCGTTGTAGAGCGTATT	RT-PCR
PA1120-F	CTGGCGGAACTCAACGAC	RT-PCR
PA1120-R	TACTTGGGTGGAGCCTCT	RT-PCR
PA4929-F	CACTGGGTATTGCCTATC	RT-PCR
PA4929-R	ATGACGATGGATGGAGTA	RT-PCR
PA0285-F	TATCTCGCAATCGCCATC	RT-PCR
PA0285-R	TCATCCAGAACACCAGTTG	RT-PCR
PA3258-F	CGAACTGGAACTACTCTG	RT-PCR
PA3258-R	GATGTTGAGGAACAGCAT	RT-PCR
PA2567-F	CGGATTTCACTCTCTACAAG	RT-PCR
PA2567-R	ACTCGGTTTCTTCCTGTT	RT-PCR
PA2072-F	GATGTATCACGCCAAGGA	RT-PCR
PA1727-R	CAGGTCATGCAGTAGTTG	RT-PCR
PA1181-F	TGAGTTCCTTCAATTACCT	RT-PCR
PA1181-R	GATCTGGTTGATGGAGTC	RT-PCR
PA4367-F	CCTATAGCGAATACTACG	RT-PCR
PA4367-R	AAGATGATTTCCGAAGTG	RT-PCR
PA4601-F	AGCCTGCTGATGAAGAAC	RT-PCR
PA4601-R	GCCTGGTAGAACTGGAAG	RT-PCR
PA4959-F	GACCGTGCAGTTCATCAA	RT-PCR
PA4959-R	CCTTGAGGATCTCCTGGTT	RT-PCR
PA3825-F	GAGCAAGTGGCGATCATG	RT-PCR
PA3825-R	AACAACGACGTGCAATAGAT	RT-PCR
PA4108-F	CTTCGCCTCGGTATGTTC	RT-PCR
PA4108-R	GAAGCCACCTCTTTTCCA	RT-PCR
PA2572-F	TCTACAACATCGGCAAGC	RT-PCR
PA2572-R	GGTCGTGGTGATAGAGCA	RT-PCR
PA4781-F	GCGTACCCATCTGCAATT	RT-PCR
PA4781-R	CGCACTTCGAGTTCCAGG	RT-PCR
PA3947-F	GAATGATTTGAATGTTCTGGTGTT	RT-PCR
PA3947-R	ACTTTCTTCAGGGCTGTGA	RT-PCR

PA5017-F	TACTCGTCACTGAGCTAC	RT-PCR
PA5017-R	GGGATGTCCTTGATGAAG	RT-PCR
PA5487-F	TGGACATCGACCACTTC	RT-PCR
PA5487-R	CAATGATCTTCAGCACCTT	RT-PCR
PA4396-F	CAATGAACGAGCAACTGG	RT-PCR
PA4396-R	TTTCCACCAGCAACCGTT	RT-PCR
PA4332-F	CATTGCAGGTGCTCGTGAT	RT-PCR
PA4332-R	GCCGCATCGCCTGTATGTA	RT-PCR
PA3311-F	AGAATTGTCGCCTGAGAG	RT-PCR
PA3311-R	AATCGTTGATCCGCTTGA	RT-PCR
PA1181-F	CATCAACAGCGACATCAC	RT-PCR
PA1181-R	ATGGAATACAGGGTCACC	RT-PCR
PA0861-F	CAACTTCCACGACTTCAG	RT-PCR
PA0861-R	CAACTTCCACGACTTCAG	RT-PCR
PA0847-F	TTACCCAGCCACGACGAT	RT-PCR
PA0847-R	CGCAGGTTGAGAGCATAGG	RT-PCR
PA0575-F	ATTTCCCGATCATCATCCTG	RT-PCR
PA0575-R	GACCTTGAGCCCGTAGAG	RT-PCR
PA0169-F	TCAGGGAGGAGAACGAAC	RT-PCR
PA0169-R	TGCTTGAAGAAGTCCACAT	RT-PCR

r: resistant.

References

1. Liberati, N.T.; Urbach, J.M.; Miyata, S.; Lee, D.G.; Drenkard, E.; Wu, G.; Villanueva, J.; Wei, T.; Ausubel, F.M. An ordered, nonredundant library of Pseudomonas aeruginosa strain PA14 transposon insertion mutants. *Proc. Natl. Acad. Sci. USA* **2006**, *103*, 2833–2838.
2. Choi, K.-H., Schweizer, H.P. Mini-Tn7 insertion in bacteria with single attTn7 sites: Example Pseudomonas aeruginosa. *Nat. Protoc.* **2006**, *1*, 153–161.
3. Fürste, J.P.; Pansegrau, W.; Frank, R.; Blöcker, H.; Scholz, P.; Bagdasarian, M.; Lanka, E. Molecular cloning of the plasmid RP4 primase region in a multi-host-range tacP expression vector. *Gene* **1986**, *48*, 119–131.
4. Weng, Y.; Chen, F.; Liu, Y.; Zhao, Q.; Chen, R.; Pan, X.; Liu, C.; Cheng, Z.; Jin, S.; Jin, Y,; et al. *Pseudomonas aeruginosa* enolase influences bacterial tolerance to oxidative stresses and virulence. *Front. Microbiol.* **2016**, *7*, 1999.
